# Intelligent Process Abnormal Patterns Recognition and Diagnosis Based on Fuzzy Logic

**DOI:** 10.1155/2016/8289508

**Published:** 2016-12-12

**Authors:** Shi-wang Hou, Shunxiao Feng, Hui Wang

**Affiliations:** ^1^School of Business, Huaihua University, Huaihua, Hunan 418000, China; ^2^School of Mechanical and Power Engineering, North University of China, Taiyuan, Shanxi 030051, China

## Abstract

Locating the assignable causes by use of the abnormal patterns of control chart is a widely used technology for manufacturing quality control. If there are uncertainties about the occurrence degree of abnormal patterns, the diagnosis process is impossible to be carried out. Considering four common abnormal control chart patterns, this paper proposed a characteristic numbers based recognition method point by point to quantify the occurrence degree of abnormal patterns under uncertain conditions and a fuzzy inference system based on fuzzy logic to calculate the contribution degree of assignable causes with fuzzy abnormal patterns. Application case results show that the proposed approach can give a ranked causes list under fuzzy control chart abnormal patterns and support the abnormity eliminating.

## 1. Introduction

Control charts are widely used in abnormity monitoring and control in manufacturing and other processes. It applies statistical signal to detect process variation, locates the assignable causes, improves the process performance, and maintains the process in satisfied quality level. For example, [1] used Shewhart control charts supplemented with runs rules to detect shifts in process variance. In [2], two stability metrics were proposed to identify underlying variation as common or special cause in biopharmaceutical processes. Reference [3] developed control charts by use of batch statistical process monitoring to perceive the process trajectory. The process variations can be divided into two categories: random and abnormal. Generally, the former is inherent in the process and is hard to eliminate; the latter indicates that there are some special sources of exceptions, which can be detected and eliminated or limited in certain range. Furthermore, the latter can be represented in control chart as a form of abnormal pattern. So it is a quick and easy way to recognize and diagnose the process by use of the abnormal signals of control chart.

However, there are uncertainties, ambiguities, and vagueness associated with the process abnormal patterns recognition and diagnosis. By considering the cause-selecting problem as a pattern classification problem, artificial neural network could deal with this problem partly in [4–7], but its internal operation mechanism cannot be obtained distinctly. If the uncertainty could be quantified to indicate the extent to which each nonrandom pattern happens and the degree to which each associated cause exists, it will facilitate the decision making. So, many researchers have introduced fuzzy logic into this field. Kahraman et al. used triangular membership functions to define various unnatural patterns in [8]. A fuzzy test was defined for each unnatural pattern using the membership value of the *i*th sample to confirm the presence of the unnatural pattern. But only simple triangular fuzzy membership functions were used to define all the patterns. Zalila et al. proposed a fuzzy supervision method for SPC which could alert operators on the process state using visual signals in [9]. These visual signals were generated using a fuzzy rule base which monitored the process center and state of dispersion. Wang and Rowlands developed a fuzzy rule based inference system based on zone rules in control charts in [10]. The input variables were the degree of membership of a point in each zone represented by fuzzy sets, and the output was the process state, mapped by eleven fuzzy If-Then rules. This approach provided improved results in terms of interpretation of data and consistency, as the numeric output from the fuzzy system indicated whether or not action should be taken, if the process was out of control. Another excellent application of fuzzy logic to control chart for individuals was developed by Tannock in [11]. In this approach, two fuzzy sets, namely, centered fuzzy set and random fuzzy set, were used. Three typical unnatural patterns: shift, Trend, and cyclical patterns were examined using these two fuzzy sets. The membership function of the centered fuzzy set was defined by the Operating Characteristic (OC) Curve of the equivalent Shewhart control chart, which considered the mean and standard deviation of the incoming distribution. The membership of the random set was determined by calculating the correlation coefficient of the series window at sample number *n* with the previous window *n* − 1. The absolute value of correlation coefficient was subtracted from unity to obtain the degree of membership, such that highly correlated data were not considered to be very random.

An investigation into the use of fuzzy logic to modify SPC rules was described in [12], with the aim of reducing the generation of false alarms and also improving the detection and detection-speed of real faults. In [13], the author proposed a neural-fuzzy model for detecting mean shifts and classifying their magnitude in multivariate process. By using a fuzzy classifier, the outputs of NN were classified into various decision intervals. Finally, the shift status was determined through an additional two-point-in-an-interval decision rule. Based on the concepts of fuzzy statistical confidence intervals and the necessity of strict dominance index, a new fuzzy control chart was proposed in [14]. In their approach, control lines of the chart were calculated as critical values of certain fuzzy statistical tests. With given significance level and necessity index, the process status was judged by the interval location relative to the control lines. Reference [15] used a fuzzy inference system to transfer the inspector's subjective rating of the products quality to a crisp number and proposed a new approach to monitoring the process when vagueness and uncertainty arise. The results showed the proposed approach could detect abnormal shifts in the process especially in small shifts.

However, all these approaches use fuzzy logic only in analyzing the control chart patterns to determine the process state. Diagnosing the assignable cause from the signals from the patterns has not been explored in these approaches.

Reference [16–18] proposed some approach to solve production problems by use of RFID-enabled technology. By attaching RFID facilities with production resources, the manufacturing process was converted into smart objects that can sense and interact. This technology can obtain the state information of nonfuzzy physical quantities. But it is still hard to realize the automatic measuring of uncertain quality attribute.

To handle the abovementioned uncertain problems, this paper proposed a fuzzy logic based inference system (FIS). The system included an input submodule, six fuzzy inference submodules, and one aggregation submodule. Each inference submodule took the characteristic numbers of control charts' abnormity pattern as input. After fuzzifying the input through respective membership function, the results were input into the corresponding inference submodules. Each submodule output its inference result by centroid defuzzifying method. In aggregation submodule, All the evidence supporting each cause from all the inference submodules were aggregated using fuzzy connective operators, and the causes were ranked according to the final aggregating results based on given fuzzy threshold.

The rest of the article is organized as follows.  Section 2 defines four basic abnormal patterns and establishes an empirical relationship model between abnormal patterns and assignable causes. Fuzzy inference system framework and design procedure are presented in Section 3. The performance of the proposed system is studied in Section 4 on the basis of an application case study.  Section 5 ends with a summary and conclusions.

## 2. Problem Description

For a controlled process, points plotted on control chart present a random pattern following normal distribution. The random state of control chart will be broken when the process is out of control, and some abnormal patterns will be shown on the control chart. These unnatural patterns can be detected by a set of rules based on the principle of small probability events. And also the causes for the current abnormity can be located based on the empirical mapping relationship between the abnormal patterns and assignable causes.

### 2.1. Control Chart Abnormal Patterns Definition

This paper considered four common abnormal patterns defined by the following rules:

1. Out of Control Limit (OCL): one or more points go beyond three sigma control limit.
2. Freak: two of three consecutive points go beyond one sigma limit on the same side of the center line.
3. Run: seven or more consecutive points are on the same side of centerline.
4. Trend: seven or more points continuously increase or decrease.

### 2.2. Empirical Mapping Relationship between Abnormal Patterns and Assignable Causes

The empirical mapping relationship between abnormal patterns and assignable causes can be built based on the nature of variation produced by the cause. According to the process abnormity implicated by control chart nonrandom pattern, assignable causes can be divided into three categories as follows (see reference [19–21]).

*(i) Isolated Causes (Denoted as C*1). Isolated causes are those that will cause a single measurement to vary drastically, resulting in one particular point falling outside the control limits producing at OCL pattern on the control chart. These causes have one-time effect. The possible causes that come under this category are as follows:

- A mistake in measurement, recording, or plotting
- Damage in handling
- Defect in raw material used for that unit alone
- False alarm

*(ii) Causes for Shift (Denoted as C*2). These causes produce a considerable shift in the process mean. These can be identified by the Freak and Run patterns on the control chart. They indicate that some event has taken place that has affected a few samples causing a drift in the mean. Usual causes that could produce this effect are as follows:

- Tool break
- Change in raw material or supplier
- Change in inspection methods or standards
- Adjustments made in machine settings
- Introduction of new workers or inspectors

*(iii) Gradual Causes (Denoted as C*3). These causes tend to change the process mean gradually over time and produce increasing or decreasing trends on the control chart and are identified by the Trend pattern. Trend patterns are produced by causes such as the following:

- Gradual introduction of new raw material
- Loosening fixtures
- Operator fatigue
- Machine tool wear

**Figure 1:**
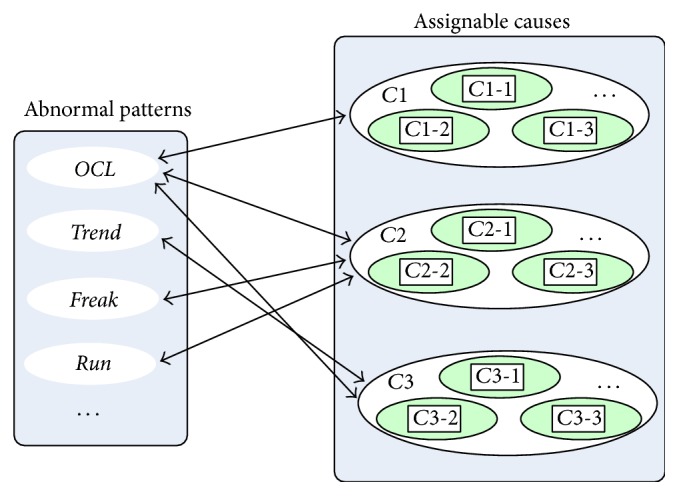
Empirical mapping relationship model of abnormal patterns and assignable causes.

So, the chart abnormal pattern-cause relationship can be modeled as shown in Figure 1.

The right nodes *C*1, *C*2, and *C*3 in assignable causes block represent isolated causes, shift causes, and gradual causes, respectively. The left nodes in abnormal patterns block represent unnatural patterns* OCL*,* Freak*,* Run*, and* Trend*. The links represent the causal relationship between the respective nodes.

Interpretation of unnatural patterns becomes complex if more than one patterns coexist. In such cases, it would again increase the list of causes to be investigated. Also, in situations such as having four points continuously increasing and fifth point falling beyond the control limit, the user would tend to interpret that as a* OCL* pattern, which actually could be a Trend pattern identified prematurely. This could mislead the assignable cause search wasting effort, time, and money. To handle such ambiguities, a fuzzy inference engine is developed which could prioritize the causes based on the unnatural patterns present.

## 3. Fuzzy Inference System (FIS) Design

### 3.1. Fuzzy Inference System Framework and Input/Output Variables Definition

**Figure 2:**
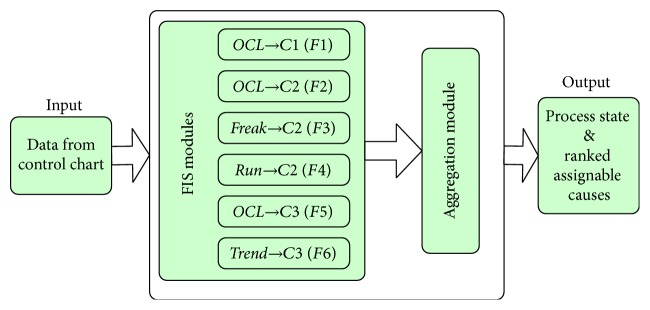
Framework of fuzzy inference system.

The components of a fuzzy inference system included input variables and its membership functions, fuzzy rule base, and output variables and its membership functions. Fuzzy inference system mapped input variables to output variables through a fuzzy rule base. The steps involved in fuzzy inference system are fuzzification, inference, and defuzzification. The input value is fuzzified by the membership functions defined for the input variables and the degree of membership is calculated. According to the chart pattern-cause relationship shown as Figure 1, each link is modeled as a separate fuzzy inference system (FIS). The proposed FIS includes six individual fuzzy inference systems as submodules and one aggregation module (as shown in Figure 2).

This paper defined four input variables named by the corresponding abnormal patters of control chart, six submodules output variables *F*1*～F*6, and three final output variables *C*1*～C*3 as follows.

*OCL*. This input variable was defined by the following formula:

(1) $$\begin{array}{c} OCL=\frac{\left|\bar{x_{i}}-\mu \right|}{\sigma }, \end{array}$$

where $\bar{x_{i}}$ denotes the *i*th sample measurement plotted on the control chart and *μ* and *σ* denote the sample mean and standard deviation, respectively.

*Freak*. The value of this variable was determined by maintaining a time-frame window of three consecutive samples and checking the point number between two times sigma line and three time sigma line within the time-frame window. The point number in [2*σ*, 3*σ*] and [−3*σ*, −2*σ*] was stored separately, and the higher number was taken as the value of* Freak*.

*Run*. Within a sample-window of seven samples, the consecutive points number above and below the center line were counted and the highest was taken as the value of* Run*.

*Trend*. By maintaining a window of seven samples and comparing each sample with the previous, the point number of consecutive increasing or decreasing in the value was recorded simultaneously, and the higher number was taken as the value of* Trend*.

*F*1*～F*6. The output variables of six fuzzy inference modules were denoted by *F*1*～F*6, valued within the range [0-1], and measured the contribution degree of corresponding assignable causes according to the occurrence degree of abnormal patterns.

*C*1*～C*3. Final output variables were denoted by *C*1*～C*3, associated with the three types of assignable causes, and their value is determined by the aggregation of *F*1*～F*6 as shown in the following part.

According to the above definition, the abnormal pattern characteristics can be obtained point by point with Algorithm 1.

Firstly, the control chart data was input to the FIS, and the characteristics numbers of four abnormal patterns all were set to zero. Every time when a new point was plotted on the control chart, the characteristic number of* OCL* was updated as their definition. For* Freak* patterns, its characteristic number was updated using the latest three successive points. The characteristic numbers of Run and Trend all were updated by use of the latest seven successive points. For* Run*,* Freak*, and* Trend* patterns, they all have two different possibilities and their characteristic numbers took the maximum of the two ways.

### 3.2. Inference Subsystem Design

This paper established the FIS by use of Matlab Fuzzy Toolbox, adopted Mamdani inference method, and defuzzified the output of FIS by centroid method.

For a given monitoring windows size, the point out of limit may appear in the start point, in the middle, or at the end of the window when some assignable causes happen. So the diagnosis about the process may be wrong only by the abnormal pattern of* OCL*. Considering the possibility that* OCL* pattern will happen under different causes, weak causal relationship was established between *OCL* and *C*2 and *C*3 in addition to the connection between *OCL* and its characteristics cause of *C*1, as shown in Figures 1 and 2.

Taking the first inference module Fis1 as example, the design process of input membership function, rule base, and the output membership functions using Matlab Fuzzy Toolbox was introduced as follows.

**Figure 3:**
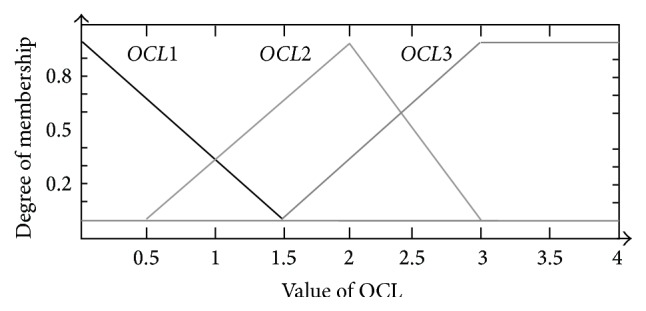
Membership function design of* OCL*.

According to the value of input variable of *OCL*, its variation was divided into three types: mild, moderate, and severe fluctuation, and each type was defined as a membership function in the process of input fuzzifing. In this manner, each input was fuzzified over predefined membership functions required by the inference rules. As for *OCL*, we defined the following three membership functions: triangular-shaped membership functions* OCL*1 (−1.5, 0, 1.5) and* OCL*2 (0.5, 2, 3) and trapezoidal-shaped membership function* OCL*3 (1.5, 3, 4, 5.5), as shown in Figure 3.

**Figure 4:**
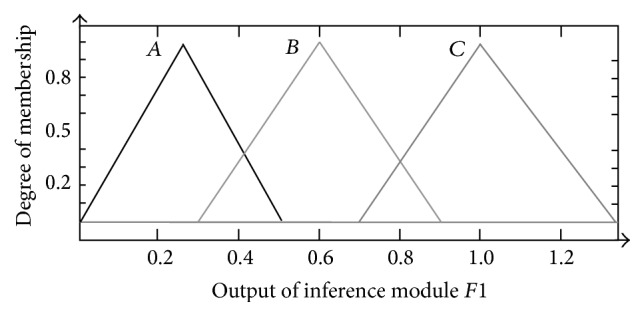
Membership function design of* F*1.

Accordingly, the output of *F*1, which denoted the contribution degree of *C*1 to *OCL*, was also defined as three triangular-shaped membership functions with different parameters,* A* (0, 0.25, 0.5),* B* (0.3, 0.6, 0.9), and* C* (0.7, 1, 1.3), respectively, to express mild, moderate, and great contribution of *C*1 to *OCL*, as shown in Figure 4.

The inference rules of *F*1 were designed by use of the inference rules editor as follows:

IF (*OCL* is* OCL*1) THEN (*F*1 is* A*)
IF (*OCL* is* OCL*2) THEN (*F*1 is* B*)
IF (*OCL* is* OCL*3) THEN (*F*1 is* C*)

**Figure 5:**
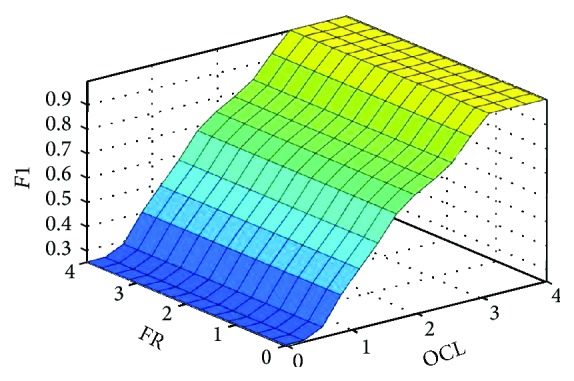
The inference surface of *F*1.

The output of subinference-module *F*1 was defuzzified by use of centroid calculation method, which returns the center of area under the curve. The defuzzified results can be viewed in Rule Viewer as Figure 5.

**Figure 6:**
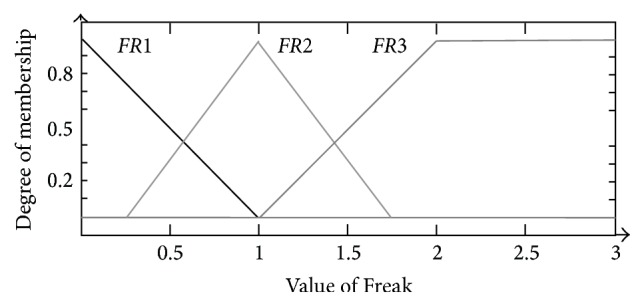
Membership function design of* Freak*.

**Figure 7:**
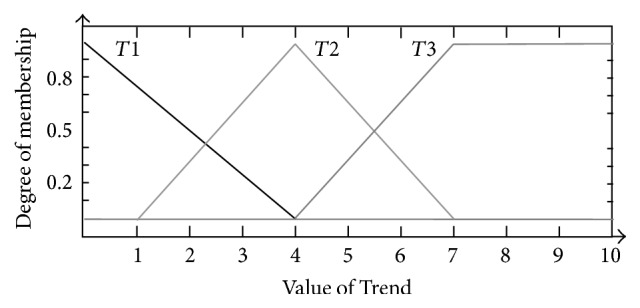
Membership function design of* Trend*.

**Figure 8:**
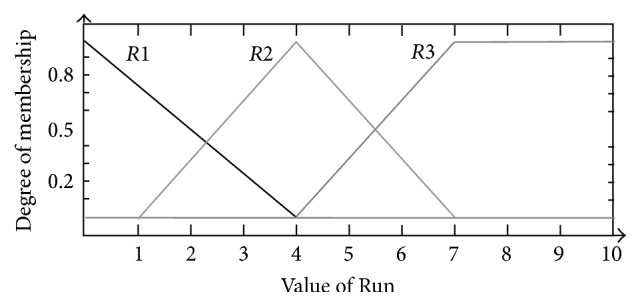
Membership function design of* Run*.

Similarly, the membership functions for* Freak*,* Trend*, and* Run* were all divided into three sections, respectively. For* Freak*, they were triangular-shaped membership functions* FR*1 (−1, 0, 1) and* FR*2 (0.25, 1, 1.75) and trapezoidal-shaped membership function* FR*3 (1, 2, 3, 4) (shown as Figure 6); for* Trend*, they were triangular-shaped membership functions* T*1 (−4, 0, 4) and* T*2 (1, 4, 7) and trapezoidal-shaped membership function* T*3 (4, 7, 10, 13) (shown as Figure 7); for* Run*, they were triangular-shaped membership functions* R*1 (−4, 0, 4) and* R*2 (1, 4, 7) and trapezoidal-shaped membership function* R*3 (4, 7, 10, 13) (shown as Figure 8).

The membership functions for* F*2 to* F*5 were the same as *F*1, that is, triangular-shaped membership functions* A* (0, 0.25, 0.5),* B* (0.3, 0.6, 0.9), and* C* (0.7, 1, 1.3) (shown as in Figure 4).

**Figure 9:**
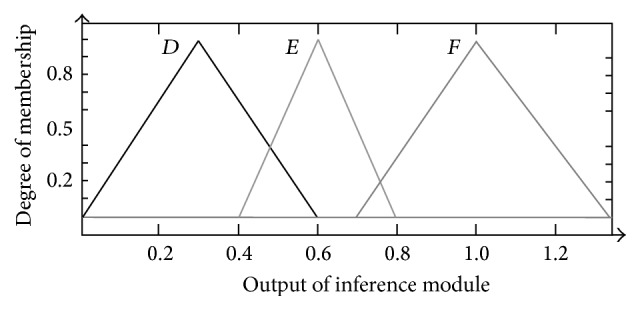
Membership function design of* F*6.

For* F*6, the membership functions were triangular-shaped membership functions* D* (0, 0.3, 0.6),* E* (0.4, 0.6, 0.8), and* F* (0.7, 1, 1.3) as shown in Figure 9.

The setting of inference rules for* F*2 to* F*6 were shown below:

FIS2: IF (*OCL* is* OCL*1) THEN (*F*2 is* A*), IF (*OCL* is* OCL*2) THEN (*F*2 is* B*), IF (*OCL* is* OCL*3) THEN (*F*2 is* C*);
FIS3: IF (*OCL* is* OCL*1) THEN (*F*3 is* A*), IF (*OCL* is* OCL*2) THEN (*F*3 is* B*), IF (*OCL* is* OCL*3) THEN (*F*3 is* C*);
FIS4: IF (*Freak* is* FR*1) THEN (*F*4 is* A*), IF (*Freak* is* FR* 2) THEN (*F*4 is* B*), IF (*Freak* is* FR* 3) THEN (*F*4 is* C*);
FIS5: IF (Run is* R*1) THEN (*F*5 is* A*), IF (*Run* is* R*2) THEN (*F*5 is* B*), IF (*Run* is* R*3) THEN (*F*5 is* C*);
FIS6: IF (Trend is T1) THEN (*F*6 is* D*), IF (*Trend* is* T*2) THEN (*F*6 is* E*), IF (*Trend* is* T*3) THEN (*F*6 is* F*).

### 3.3. Design of Fuzzy Aggregation Operator

For each type of assignable cause, its contribution degree to current control chart abnormity depends on the following two factors:

1. Own characteristic patterns
2. Other patterns except its characteristic patterns

So the final contribution degree result of *C*1 to *C*3 was aggregated by two steps. In the first step, all the inference module output of characteristic patterns was aggregate by operator of Max. As described in Section 3.1,* OCL* was the characteristic pattern of *C*1,* Freak* and* Run* were the characteristic patterns of *C*2, and* Trend* was the characteristic pattern of *C*3. In the second step, the results of the first step were aggregated further with the inference module output of noncharacteristic pattern using algebraic sum operator of ⊕, whose operational rule was defined as follows:

(2) $$\begin{array}{c} a⊕b=a+b-a*b, \end{array}$$

where the symbols *a* and *b* mean two numbers of membership degree.

The final output results of *C*1*～C*3 were denoted as follows:

(3) C1=F1,

(4) C2=max⁡F4,F5⊕F2,

(5) C3=F6⊕F3.

The work principle of FIS is described as follows. When data was plotted in control chart, the characteristic number of four predefined abnormal patterns is calculated and then their occurrence degree is calculated by use of the corresponding input membership functions. Then, according to the related inference rules with some patterns, that is, Fis1~Fis6, the contribution degree of some causes to the current abnormal patterns are obtained. Then, these values are aggregated by use of formulae (3)~(5). Finally, the assignable causes of *C*1*～C*3 were ranked according to the value of the aggregated results that represented the contribution degree of *C*1*～C*3 to current abnormity showing in control chart. By setting trigger thresholds *α*, *β*, and *γ* for three causes, the malfunction elimination will be carried out once some aggregated result is larger than the corresponding threshold; that is, *C*1 ≥ *α*, *C*2 ≥ *β*, or *C*3 ≥ *γ*.

## 4. Case Study

This paper took a roller production workshop of textile machinery factory as example, collected the quality inspecting data of some sampling period, and plotted them in control chart to validate the proposed approach.

The specification center of roller parts is 35 mm, the sample size is *N* = 72, and the number of subgroups *n* = 6. Perform the following tests for special causes:

- 1 point > 3 standard deviations from center line
- 7 points in a row on the same side of center line
- 7 points in a row, all increasing or all decreasing
- 2 out of 3 points > 2 standard deviations from center line (same side)

**Figure 10:**
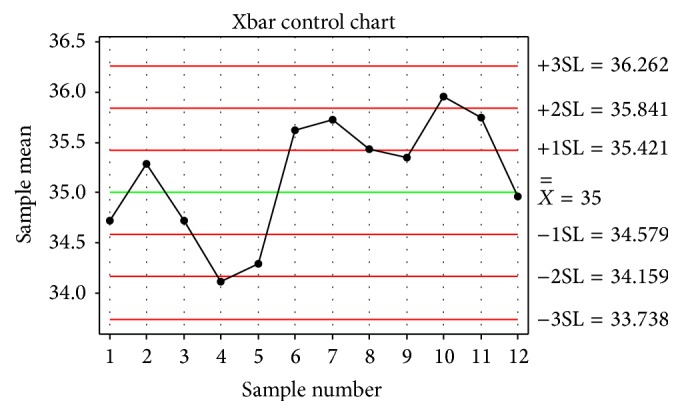
Xbar chart of roller quality inspecting data.

The sample data was plotted in control chart by Minitab software, as shown in Figure 10. There were no abnormal points that violated the testing rules. So the process was in control and needed no adjustment according to the conventional control chart application principle.

**Figure 11:**
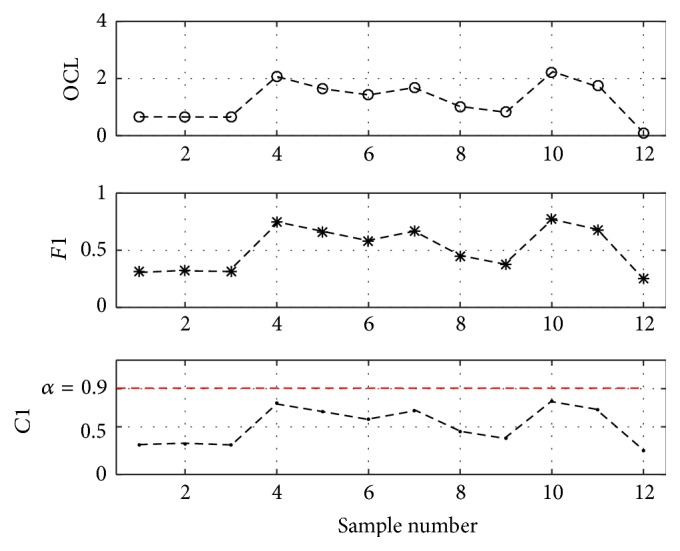
Contribution degree of *C*1.

**Figure 12 fig12:**
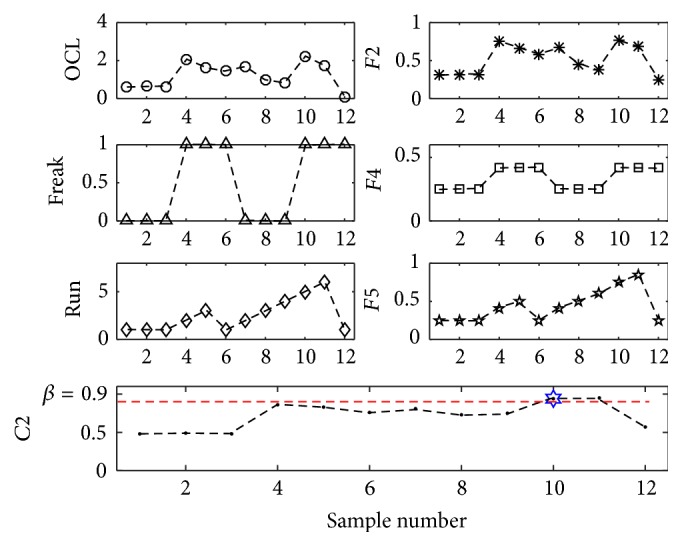
Contribution degree of *C*2.

**Figure 13:**
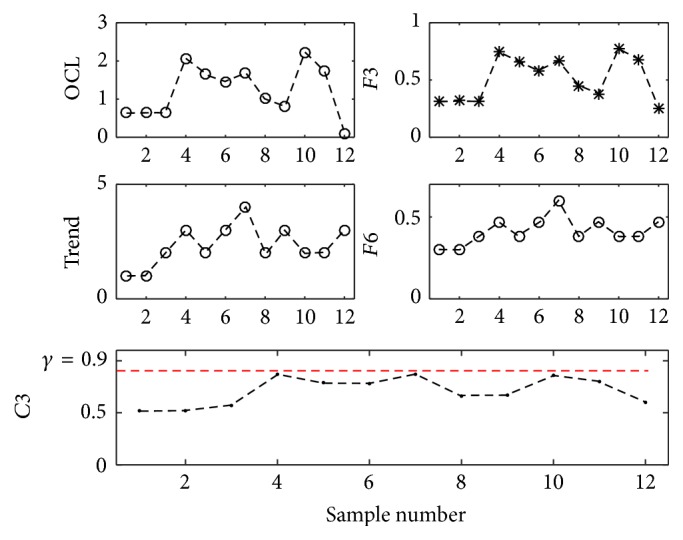
Contribution degree of *C*3.

Set *α* = *β* = *γ* = 0.9 and carry out the monitoring and diagnosing process by use of the proposed FIS. The results were shown in Figures 11 [Fig fig12]–13.

According to formula (3)~(5), Figure 11 shows the contribution degree of *C*1, that is,* F*1, to current pattern of* OCL*, Figure 12 shows the contribution degree of *C*2, that is, max⁡(*F*4, *F*5) ⊕ *F*2, to current pattern of* OCL*,* Freak*, and* Run*, Figure 13 shows the contribution degree of *C*3, that is, *F*6 ⊕ *F*3, to current pattern of* OCL* and* Trend*.

As seen from Figure 12, when the tenth point was plotted in control chart, the output *C*2 of FIS reached 0.94 and was larger than the set value *β* = 0.9 of *C*2. According to formula (4), this result was produced by Fis5 and Fis2 and the potential abnormal patterns were* Run* and* OCL*. Since *C*2 ranked first and also trigged the threshold, and the elimination work was started with *C*2 causes, which included tools condition, change of raw material, and machine setting adjustment, and so forth. After abnormity eliminating, the following 12 sample data pieces were plotted in control chart and there was no abnormity found by use of the proposed FIS.

For Figures 11 and 13, there are no sample points that triggered the presupposed threshold, so the process needs no adjustment.

Before the introduction of the threshold, the eliminating work is started only when some aggregated result reached 1. For the control chart as Figure 6, there was no aggregated result that reached 1 seen from Figures 11 [Fig fig12]–13, and so the process was not adjusted. As a result, the potential and fuzzy abnormal patterns can not be identified and prevented from progressing.

## 5. Conclusions

This paper proposed a fuzzy inference system to deal with the abnormal information on control chart. The potential abnormal patterns were quantitatively described point by point using fuzzy membership. The contribution degree of each assignable cause was determined and ranked in terms of the occurrence degree of abnormal patterns and fuzzy inference rules. Once the contribution degree was greater than the set threshold *α* (*β* or *γ*), the corresponding cause will be inspected as the main object. It will accelerate the abnormity diagnosis and recovery process and reduce process running time in abnormal state. Application results show that the proposed FIS system has good effect on the fuzzy abnormal patterns that conventional quality control tool can not recognize.

This paper analyzed the proposed system based on XBar chart and further research can be extended to other types of control chart and multivariate control charts. As for the interior of each category of assignable causes, we can analyze the contribution degree of each detail causes according to the quality control experience of specific process, so the system can provide a concrete and direct abnormity eliminating approach. Also, the setting of threshold value for different conditions may be worth pursuing in future research.

## Figures and Tables

**Algorithm 1:**
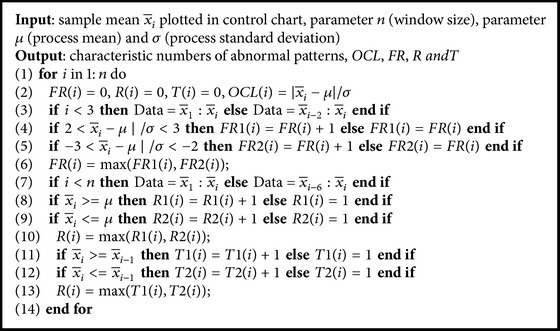
Calculating abnormal pattern characteristics.
